# *Morchella esculenta* cultivation in fallow paddy fields and drylands affects the diversity of soil bacteria and soil chemical properties

**DOI:** 10.3389/fgene.2023.1251695

**Published:** 2023-09-12

**Authors:** Mingzheng Duan, Chengcui Yang, Liuyuan Bao, Duo Han, Huaizheng Wang, Yongzhi Zhang, Honggao Liu, Shunqiang Yang

**Affiliations:** ^1^ Yunnan Key Laboratory of Gastrodia Elata and Fungal Symbiotic Biology, College of Agronomy and Life Sciences, Zhaotong University, Zhaotong, China; ^2^ Yunnan Engineering Research Center of Green Planting and Processing of Gastrodia Elata, College of Agronomy and Life Sciences, Zhaotong University, Zhaotong, China

**Keywords:** rice, corn, soils, 16S rRNA, metabarcoding, NPK

## Abstract

The properties of paddy field (DT) and dry land (HD) soil and food production can be enhanced by the cultivation of *Morchella esculenta* (ME) during the fallow period. However, whether ME cultivation affects the soil health and microbial diversity of paddy fields and drylands during the cultivation period remains unclear, and this has greatly limited the wider use of this cultivation model. Here, we analyzed the soil chemical properties and bacterial diversity (via metabarcoding sequencing) of DT and HD soils following ME cultivation. Our findings indicated that ME cultivation could enhance soil health. The content of soil phosphorus and potassium (K) was increased in DT soil under ME cultivation, and the K content was significantly higher in HD soil than in DT soil under ME cultivation. ME cultivation had a weak effect on alpha diversity, and ME cultivation affected the abundance of some genera of soil bacteria. The cultivation of ME might reduce the methane production capacity of DT soil and enhance the nitrogen cycling process of HD soil based on the results of functional annotation analysis. Network analysis and correlation analysis showed that *Gemmatimonas*, *Bryobacter*, and *Anaeromyxobacter* were the key bacterial genera regulating soil chemical properties in DT soil under ME cultivation, and *Bryobacter*, *Bacillus*, *Streptomyces*, and *Paenarthrobacter* were the key taxa associated with the accumulation of K in HD soil. The results of our study will aid future efforts to further improve this cultivation model.

## 1 Introduction

The sustainable production of food has long been a major goal for mankind, and this is being increasingly challenged by human population growth and ongoing climate change (Zhang et al., 2018). Thus, much effort has been made to enhance the efficiency of land use and increase food production (Brown et al., 2014; Di Benedetto et al., 2017; Babu et al., 2020).

*Morchella esculenta* (i.e., Yang-Du-Jun (YDJ); ME) is an economically valuable edible fungus with beneficial medicinal properties (Paul et al., 2018; Sunil and Xu, 2022). ME can be cultivated in both paddy field (DT) and dryland (HD) environments. ME has often been cultivated during the fallow period in both DT and HD environments to enhance food production.

Soil health is affected by various biotic and abiotic factors; microbes, especially bacteria and fungi, play a critically important role in soil biogeochemical cycles and the conversion of soil nutrients (Basu et al., 2021). The cultivation of mushrooms is thought to have a substantial effect on soil health. For example, the growth of *Leucocalocybe mongolica* mycelium in soil in a fairy ring ecosystem has been shown to increase soil nutrient abundance (Duan et al., 2022a); this can in turn enhance plant growth, increase the abundance of flavonoid metabolites, and promote hormone synthesis (Duan et al., 2022b). The cultivation of *Dictyophora indusiata* inhibits the activity of soil urease, which reduces the environmental loss of soil N (Duan et al., 2023a). Therefore, the cultivation of macrofungi (mushrooms) has a major effect on soil ecology.

Several previous studies have examined the effects of different *D. indusiata* cultivation techniques on soil bacterial diversity (Orlofsky et al., 2021; Zhang et al., 2023). However, no studies to date have examined the effects of ME cultivation on soil health and the diversity of soil microbes in DT and HD during the fallow period.

The diversity of soil bacteria plays a more important role in mediating the soil-ecological effects of macrofungi than the diversity of soil fungi, and this is related to the fact that the biomass of soil bacteria is much higher than that of fungi (Duan and Bau, 2021). Sequencing technology is one of the most effective approaches for measuring the diversity of soil microbes (Nesme et al., 2016). The diversity of soil microbes in various types of soil has been analyzed in previous studies using metabarcoding sequencing, including grassland soil (Duan et al., 2021), sugarcane field soil (Duan et al., 2022c; Duan et al., 2023b), and soil under ME cultivation (Orlofsky et al., 2021; Zhang et al., 2023).

Although previous studies have demonstrated that the cultivation of ME during fallow periods in DT and HD can enhance food production, the effects of ME cultivation on soil ecology and the abundances of soil nutrients remain unclear, and this greatly limits the use of this cultivation model. An improved understanding of the effects of ME cultivation on the chemical properties of soil and the bacterial diversity of soil in DT and HD is essential for improving the effectiveness of this cultivation model.

Here, we cultivated ME during the fallow periods in DT and cornfields and collected soil samples at harvest. Soil chemical analysis and metabarcoding sequencing were conducted to analyze the effects of ME cultivation on changes in soil nutrients and bacterial diversity and functions. In addition, we analyzed associations between changes in bacterial populations and the content of soil nutrients under ME cultivation.

## 2 Materials and methods

### 2.1 Materials

**FIGURE 1:**
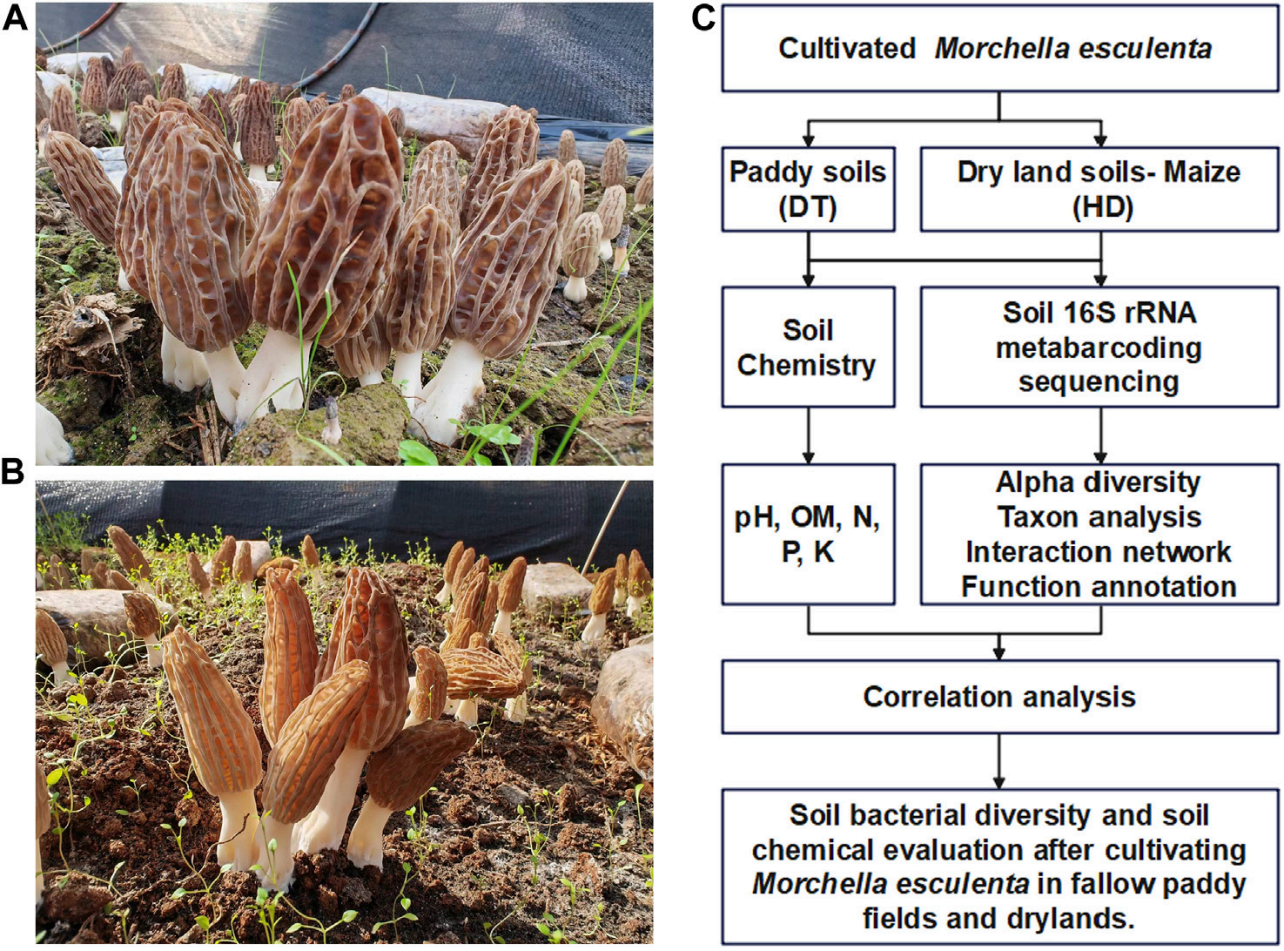
Experimental design for the cultivation of ME in paddy fields and dry lands during the fallow period. **(A)** ME cultivated in paddy soil (DT soils). **(B)** ME cultivated in dryland soils (HD soils). **(C)** Technical outline of this study.

DT (Figure 1A) and HD (Figure 1B) sampling sites were located in Ludian County, Zhaotong City, Yunnan Province, China. ME (Variety name: Liu-mei, obtained from Longxing Biotechnology Co., LTD, China.) was cultivated in October 2021 and harvested in January 2022. The yield of ME in DT and HD filed was 504 kg/km^2^ and 210 kg/km^2^.

### 2.2 Methods

#### 2.2.1 Field sampling method

Soil was collected from under the ME fruiting bodies; samples were obtained from 10 random points and mixed and divide it into 3 portions as biological repetitions for subsequent analysis (including the DT-YDJ and HD-YDJ treatments). Sampling sites in the same area where ME was not cultivated were used as the control groups (including the DT-CK and HD-CK treatments). Soil samples were collected from the top layer at a depth of 0–10 cm. The soil was then sieved through a 0.425 mm filter, packed into sterile cryostorage tubes, and frozen in liquid N for analysis of the metabarcoding sequencing data. The soil samples used in analyses of soil chemical properties were air-dried and stored at ambient temperature.

#### 2.2.2 Analysis of soil chemical properties

Soil pH levels are detected using the potentiometry method (Food and Agriculture Organization, 2007). The content of available N in soil was determined using the Kjeldahl method with sulfuric acid–accelerator digestion (Food and Agriculture Organization, 2015). The content of available P in soil was determined via NaOH alkali melting and molybdenum–antimony resistance spectrophotometry (New south wales, 1988a). The content of available K in soil was determined via NaOH alkali melting and flame photometry (New south wales, 1988b). The K-dichromate oxidation external heating method was used to determine the content of soil organic matter (OM) (Food and Agriculture Organization, 2006).

#### 2.2.3 Metabarcoding analysis

DNA was extracted from soil samples (3 g) using a HiPure Soil DNA Kit (Magen, Guangzhou, China) per the manufacturer’s instructions following a previously described method (Duan et al., 2022b). The primers 341F (5′-CCTACGGGNGGCWGCAG-3′) and 806R (5′-GGACTACHVGGGTATCTAAT-3′) were used to amplify the 16S rDNA V3-V4 region in the ribosomal RNA gene for bacteria via polymerase chain reaction (PCR) (Guo et al., 2017). The purified amplicons, which were pooled in equimolar ratios per the standard protocol, were paired-end sequenced using an Illumina Novaseq 6000 platform. The average sequencing depth of each sample is greater than 100000 tags. The UPARSE (Edgar, 2013) (version 9.2.64) pipeline was used to cluster the clean tags into operational taxonomic units (OTUs) with at least 97% similarity. The most abundant tag sequence was selected as the representative sequence within each cluster. The representative OTU sequences were classified using a naïve Bayesian model with The Ribosomal Database Project classifier (version 2.2) (Wang et al., 2007) based on the SILVA (16S rRNA OTUs) database (Pruesse et al., 2007) (version138.1) with a confidence threshold value of 0.8. R software was used to create all figures. Comparisons of OTUs among groups were made using the VennDiagram package (version 1.6.16) in R software (Chen and Boutros, 2011). Nonmetric multidimensional scaling (NMDS) analysis and analysis of the Shannon index were conducted to measure between-group variance. QIIME (version 1.9.1) software was used to calculate the observed species (Sob), Shannon, and Good’s coverage indexes (Caporaso et al., 2010). After the OTU numbers were log_2_-transformed, NMDS was conducted in the vegan package (version 2.5.3; http://CRAN.R-project.org/package=vegan; 2022.11.9) in R software to characterize variation in the composition of OTUs among experimental groups. The VennDiagram package (version 1.6.16) in R was used to conduct a Venn analysis that compared the OTUs among the different groups (Chen and Boutros, 2011). Linear discriminant analysis effect size (LEfSe) software was used to conduct LEfSe analysis (Segata et al., 2011), and the value of the linear discriminant analysis (LDA) filtrate score was 4. The ecologically relevant functions of bacteria were predicted using the functional annotations of the prokaryotic taxa (FAPROTAX) database (version 1.0) (Louca et al., 2016) and BugBase database (Ward et al., 2017). OmicShare tools, a dynamic, real-time, and interactive online platform for data analysis (http://www.omicsmart.com) (accessed on 12 April 2023), was used to construct networks based on the correlation coefficients.

#### 2.2.4 Correlation analysis

The OmicShare tools platform was used to analyze the correlation between the metabarcoding data and soil chemical properties data. Data on the soil chemical properties and OTUs were log_10_ transformed. Correlations were evaluated using the soil chemical properties and the numbers of the 20 most abundant bacterial genera. The correlation heat map tools in OmicShare (accessed on 12 April 2023) were used to make a heat map based on the Pearson correlation coefficients.

## 3 Results and discussion

### 3.1 ME cultivation can affect soil chemical properties

**FIGURE 2:**
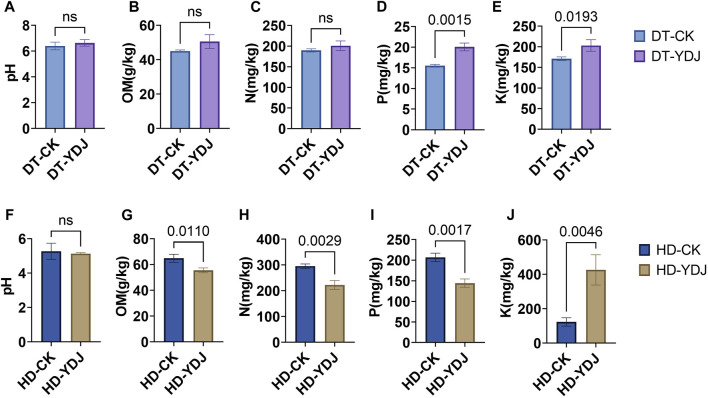
Analysis of soil chemical properties following ME cultivation. The abscissa shows the control group (CK) and treatment group (YDJ), and the ordinate shows the chemical properties and their units. DT stands for paddy soil, and HD stands for cornfield soil. **(A, F)** show the pH, **(B, G)** show the content of organic matter (OM), **(C, H)** show the content of hydrolyzable N, **(D, I)** show the content of available P, and **(E, J)** show the content of available K. “ns” stands for not significant, and the number on the bar indicates the *p*-value.

We analyzed the pH and abundances of OM, N, P, and K in both DT and HD soils under ME cultivation. In DT soils, no significant differences were observed between the CK and YDJ treatments in the pH (6.4–6.6; Figure 2A), content of OM (45–50.5 g/kg; Figure 2B), and content of N (189.6–201.1 mg/kg; Figure 2C). However, the content of P (15.5–20.1 mg/kg; Figure 2D) and K (170.9–203 mg/kg; Figure 2E) was significantly higher in the YDJ treatment than in the CK treatment (P content: *p* = 0.0015; K content: *p* = 0.0193). Therefore, although no significant differences in pH, OM, and N were observed between the CK and YDJ treatments in DT soils, the content of P and K was markedly higher in the YDJ treatment than in the CK treatment, indicating that ME cultivation increased the availability of these nutrients in the soil. No significant differences in the pH of HD soils (5.1–5.2; Figure 2F) were observed between the CK and YDJ treatments. However, the content of OM (55.5–64.9 g/kg; Figure 2G), N (221.8–295 mg/kg; Figure 2C), and P (144.3–206.6 mg/kg; Figure 2I) was significantly higher in the CK treatment than in the YDJ treatment (OM content: *p* = 0.011; N content: *p* = 0.0029; P content: *p* = 0.0017), and the content of K (123–426.2 mg/kg; Figure 2J) was significantly higher in the YDJ treatment than in the CK treatment (*p* = 0.0046). There were no significant differences in pH in HD soils between the CK and YDJ treatments, and the content of OM, N, and P was markedly higher in the CK treatment than in the YDJ treatment; the content of K was higher in the YDJ treatment than in the CK treatment, suggesting that the effects of ME cultivation on the availability of nutrients soil vary among soil types. Previous studies have indicated that the cultivation of ME with peach trees and ME fermentation with cultivated maize can increase the content of effective K in the soil (Phanpadith et al., 2020; Song et al., 2021), which is consistent with the results of this study. Similar findings were observed in DT and HD soil, and the effect in HD soil was significant. This indicates that ME cultivation can be used as a replacement for some of the positive effects of K fertilizer. Previous studies have shown that interactions between large fungi and soil can increase soil fertility (Duan et al., 2022a; Duan et al., 2023a), which stems from the fact that interactions of fungi with soil can alter chemical properties, soil enzyme activities, ion concentrations, and other factors; stress resistance can be enhanced via interactions with plants (Duan et al., 2022a; Duan et al., 2022b; Duan et al., 2023a). Overall, the results of our study indicate that the cultivation of ME during the fallow periods in DT and HD not only increases the utilization efficiency of farmland but also possibly improving soil nutrient levels.

### 3.2 Sequencing statistics and alpha diversity of DT and HD soils under ME cultivation

**FIGURE 3:**
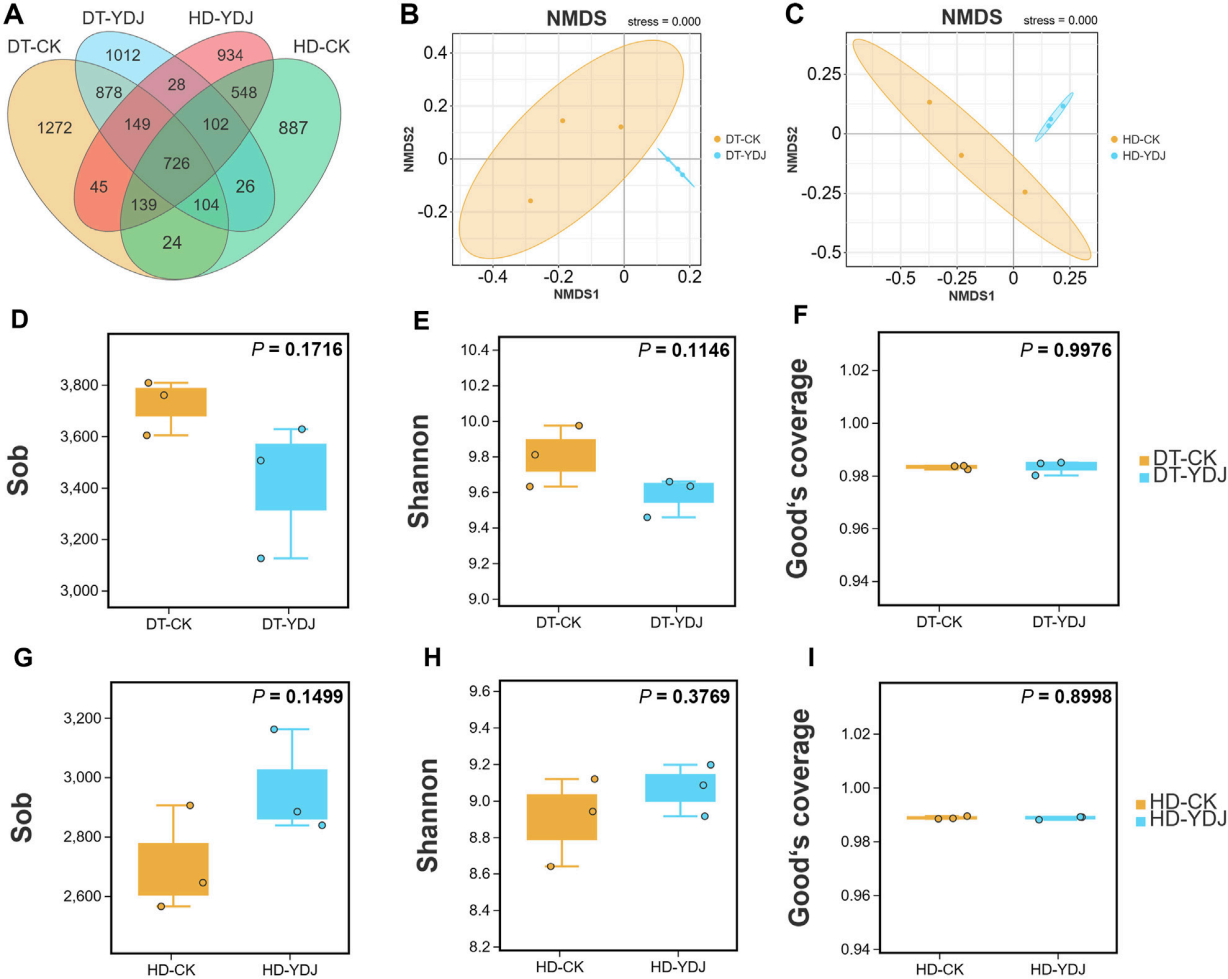
Venn map, NMDS analysis, and alpha diversity of DT and HD soils under ME cultivation. **(A)** Venn analysis of bacterial OTUs. **(B, C)** NMDS analysis of the bacterial OTUs in the DT-CK/YDJ and HD-CK/YDJ treatments. The colored dots in the figures indicate the different sample groups. **(D, G)** show observed species values, **(E, H)** show Shannon index values, and **(F, I)** show Good’s coverage index values. The abscissa shows the CK and YDJ treatments in DT and HD soils, and the ordinate shows the alpha diversity indexes and their units. *p*-values were calculated by *t*-tests.

We conducted a metabarcoding survey to characterize the diversity of soil bacteria under ME cultivation. 16S rRNA sequencing yielded 1,450,023 effective metabarcoding tags. A total of 3,202 bacterial OTUs were identified per sample on average according to OTU clustering analysis of the soil samples (Table 1). A Venn diagram of the OTUs revealed that 726 (10.56%) of the 6,874 bacterial OTUs were present in all soil samples, and 1,012 (14.72%) and 934 (13.58%) of the OTUs were unique to the DT-YDJ and HD-DYJ treatments, respectively (Figure 3A). NMDS plots based on the abundances of OTUs revealed separation in bacterial OTUs between the DT-CK/YDJ (Figure 3B) and HD-CK/YDJ FR (Figure 3C) treatments, indicating that the distribution of bacteria in these treatments significantly differed. We also compared the alpha diversity among samples using a t-test. The mean values of the Sob index of soil samples were 3,723 and 3,419 in the DT-CK and DT-YDJ treatments, respectively (Figure 3D); the mean values of the Sob index were 2,705 and 2,691 in the HD-CK and HD-YDJ treatments, respectively (Figure 3G). The mean values of the Shannon index were 9.8 and 9.58 in the DT-CK and DT-YDJ treatments, respectively (Figure 3E); the mean values of the Shannon index were 8.89 and 9.06 in the HD-CK and HD-YDJ treatments, respectively (Figure 3H). All values of the Good’s coverage index were greater than 0.98, which indicates that the sequencing depth of all samples was sufficient. However, the effect of ME cultivation on the alpha diversity index of soil bacteria was weak in both DT and HD soil, and none of the differences between all comparison groups were significant (*p* > 0.05). A previous metabarcoding study of soil under ME cultivation at different levels of maturity revealed that around 450 of OTUs were identified, and the Shannon diversity index of soil samples was between 4.8 and 5.1 (Orlofsky et al., 2021). The number of OTUs and Shannon index values were much lower in our study than in this previous study. The Shannon index values of soil at different maturity levels ranged from 4 to 6 in another metagenomic sequencing study of soil under ME cultivation at all maturity stages, and this significantly differs from the results of our study (Zhang et al., 2023). This might stem from differences in soil type among studies; it also suggests that OTU richness and alpha diversity might not be the primary factors underlying the interactions between ME and soil. The fact that we did not detect any differences in alpha diversity between soils under ME cultivation and control soil (i.e., YDJ and CK treatments, respectively) provides additional support for this finding.

**TABLE 1:** Bacterial metabarcoding sequencing data.

ID	Clean tags	N90	OTUs
DT-CK-1	124737	441	3603
DT-CK-2	127432	441	3807
DT-CK-3	119486	441	3759
DT-YDJ-1	97653	441	3125
DT-YDJ-2	126552	441	3505
DT-YDJ-3	127208	441	3627
HD-CK-1	115814	441	2565
HD-CK-2	123901	441	2905
HD-CK-3	115817	441	2645
HD-YDJ-1	129155	441	3161
HD-YDJ-2	122425	441	2884
HD-YDJ-3	119843	441	2838

### 3.3 Composition of soil bacterial taxa

**FIGURE 4:**
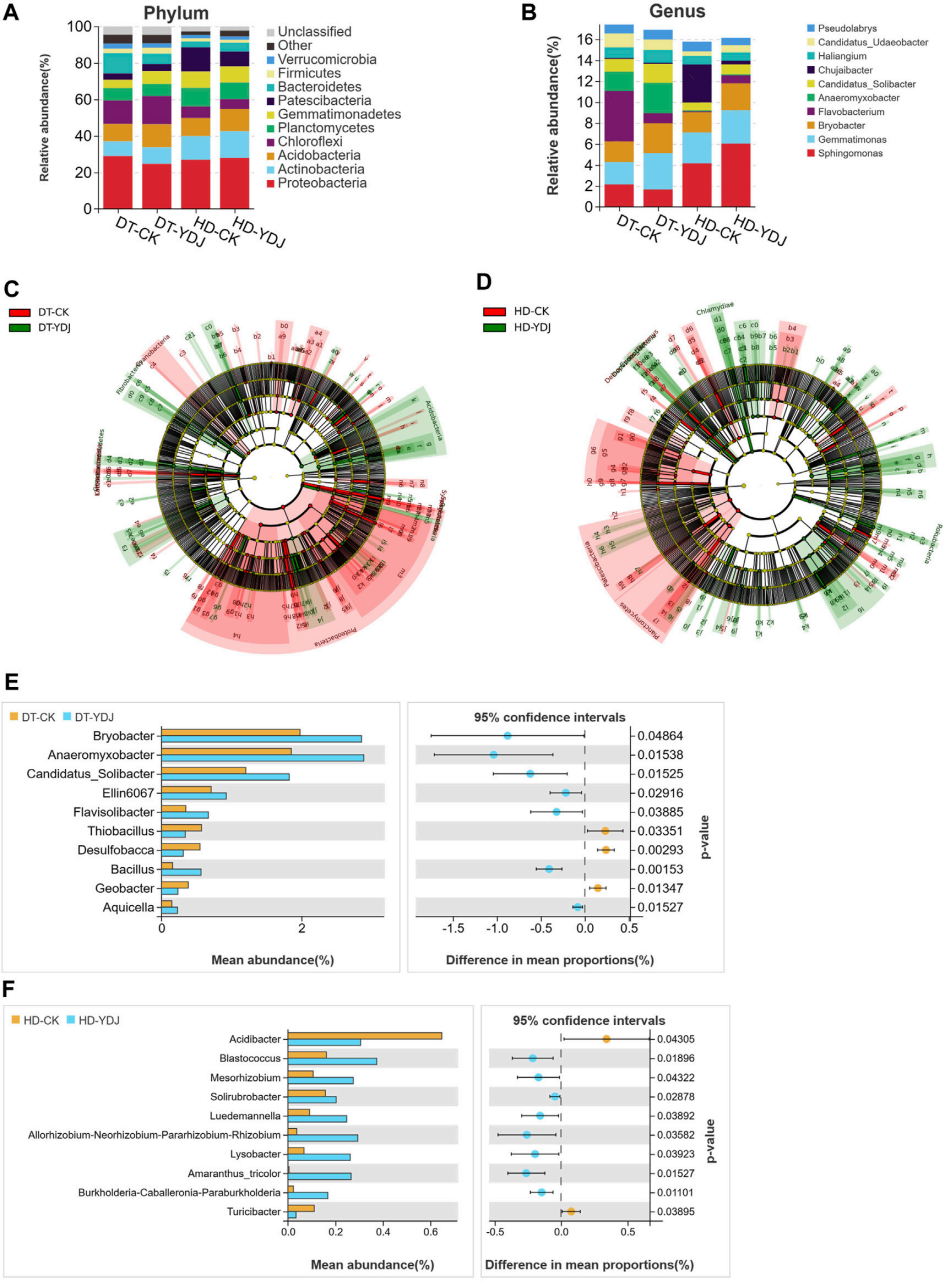
Analysis of soil bacterial taxa in DT and HD soils under ME cultivation. The relative abundance of bacterial **(A)** phyla and **(B)** genera. The *x*-axis indicates the different treatments, the *y*-axis indicates the relative abundances of bacterial phyla or genera, and the different colors correspond to different phyla or genera. The legend is shown on the right side of the figure. LEfSe analysis of **(C)** DT soils and **(D)** HD soils under ME cultivation. The taxon names represented by specific symbols can be found in Supplementary Figure S1, S2. **(E, F)** indicate the analysis of differentially abundant genera in DT and HD soils following ME cultivation. The vertical axis on the left half of the graph shows differentially abundant species, and the horizontal axis shows mean species abundance. On the right half, the horizontal axis shows the difference in abundance between groups, and the color of the dots indicates the group with higher abundance. The error bars of the dots indicate fluctuations in the 95% confidence interval of the difference. The vertical axis indicates the significance of the difference between corresponding species groups (i.e., the magnitude of the *p*-value).

We analyzed differences in the composition of the bacterial community between DT and HD soils under ME cultivation based on the SILVA database. The five most abundant bacterial phyla (Figure 4A) were Proteobacteria (28.99%, 24.68%, 26.97%, and 28.01% in the DT-CK, DT-YDJ, HD-CK, and HD-YDJ treatments, respectively), Actinobacteria (8.07%, 9.07%, 12.96%, and 14.57% in the DT-CK, DT-YDJ, HD-CK, and HD-YDJ treatments, respectively), Acidobacteria (9.48%, 12.62%, 9.88%, and 12.15% in the DT-CK, DT-YDJ, HD-CK, and HD-YDJ treatments, respectively), Chloroflexi (12.87%, 15.44%, 6.39%, and 5.41% in the DT-CK, DT-YDJ, HD-CK, and HD-YDJ treatments, respectively), and Planctomycetes (6.82%, 6.65%, 10.12%, and 9.11% in the DT-CK, DT-YDJ, HD-CK, and HD-YDJ treatments, respectively); the five most abundant bacterial genera (Figure 4B) were *Sphingomonas* (2.15%, 1.66%, 4.15%, and 6.04% in the DT-CK, DT-YDJ, HD-CK, and HD-YDJ treatments, respectively), *Gemmatimonas* (2.12%, 3.46%, 2.94%, and 3.19% in the DT-CK, DT-YDJ, HD-CK, and HD-YDJ treatments, respectively), *Bryobacter* (1.98%, 2.85%, 1.94%, and 2.56% in the DT-CK, DT-YDJ, HD-CK, and HD-YDJ treatments, respectively), *Flavobacterium* (4.8%, 0.95%, 0.08%, and 0.76% in the DT-CK, DT-YDJ, HD-CK, and HD-YDJ treatments, respectively), and *Anaeromyxobacter* (1.85%, 2.89%, 0.08%, and 0.76% in the DT-CK, DT-YDJ, HD-CK, and HD-YDJ treatments, respectively). LEfSe analysis revealed that Acidobacteria (log_10_ LDA score >3, *p* < 0.05) and Gemmatimonadetes (log_10_ LDA score >3, *p* < 0.05) were the marker phyla in the DT-YDJ vs. DT-CK comparison group (Figure 4C). Cyanobacteria (log_10_ LDA score >3, *p* < 0.05) and Chlamydiae (log_10_ LDA score >3, *p* < 0.05) were identified as the marker phyla in the HD-YDJ vs. HD-CK comparison group (Figure 4D). Acidobacteria was enriched in both DT and HD soils under ME cultivation (i.e., DT-YDJ and HD-YDJ treatments); it was also the most abundant bacterial phylum in the DT-YDJ treatment, suggesting that ME cultivation has the most pronounced effect on the abundance of Acidobacteria. Acidobacteria is one of the most common bacteria in soil. It can survive in different soil environments; play a key role in maintaining the structure and function of soil microbial communities, and mediate the conversion of soil nutrients (Lauber et al., 2009; Kielak et al., 2016). Thus, the cultivation of ME in DT and HD soils can enhance soil health by promoting the proliferation of Acidobacteria. Next, we identified bacterial genera that showed significant differences in abundance between DT and HD soils using *t*-tests. In DT soils (Figure 4E), *Bryobacter* (relative abundance of 1.98% and 2.85% in the DT-CK and DT-YDJ treatments, respectively; *p* = 0.04), *Anaeromyxobacter* (1.85% and 2.89% in the DT-CK and DT-YDJ treatments, respectively; *p* = 0.01)*, Candidatus_Solibacter* (1.20% and 1.82% in the DT-CK and DT-YDJ treatments, respectively; *p* = 0.01)*, Ellin6067* (0.70% and 0.92% in the DT-CK and DT-YDJ treatments, respectively; *p* = 0.01), and *Bacillus* (0.15% and 0.56% in the DT-CK and DT-YDJ treatments, respectively; *p* = 0.001) were the five most differentially abundant genera; in HD soils (Figure 4F), *Acidibacter* (relative abundance of 0.64% and 0.30% in the HD-CK and HD-YDJ treatments, respectively; *p* = 0.04), *Blastococcus* (0.16% and 0.37% in the HD-CK and HD-YDJ treatments, respectively; *p* = 0.01)*,* and *Mesorhizobium* (0.10% and 0.27% in the HD-CK and HD-YDJ treatments, respectively; *p* = 0.04) were the three most differentially abundant genera. In which, a study has shown that *Bryobacter* and *Candidatus_Solibacter* in rhizosphere soil has the potential to regulate *Cinnamomum migao* metabolism (Li et al., 2023); *Ellin6067* as a kind of nitrosifying bacteria, which may participate in the soil nitrogen cycling process (Lv et al., 2022); *Bacillus* is a well-known plant growth-promoting bacterium (Sheng, 2005; Ali et al., 2021). *Blastococcus* with potential capacity improve the soil nutrient supply (Li et al., 2022); *Mesorhizobium* be found with capacity of improves chickpea growth under chromium stress and alleviates chromium contamination of soil (Naz et al., 2023). Our findings indicate that the composition of the bacterial community differed significantly in DT and HD soils under ME cultivation. The relative abundances of the top bacterial phyla and genera differed between the two soil types, suggesting that the soil type has a major effect on the bacterial community.

### 3.4 Functional annotation of soil bacteria

**FIGURE 5:**
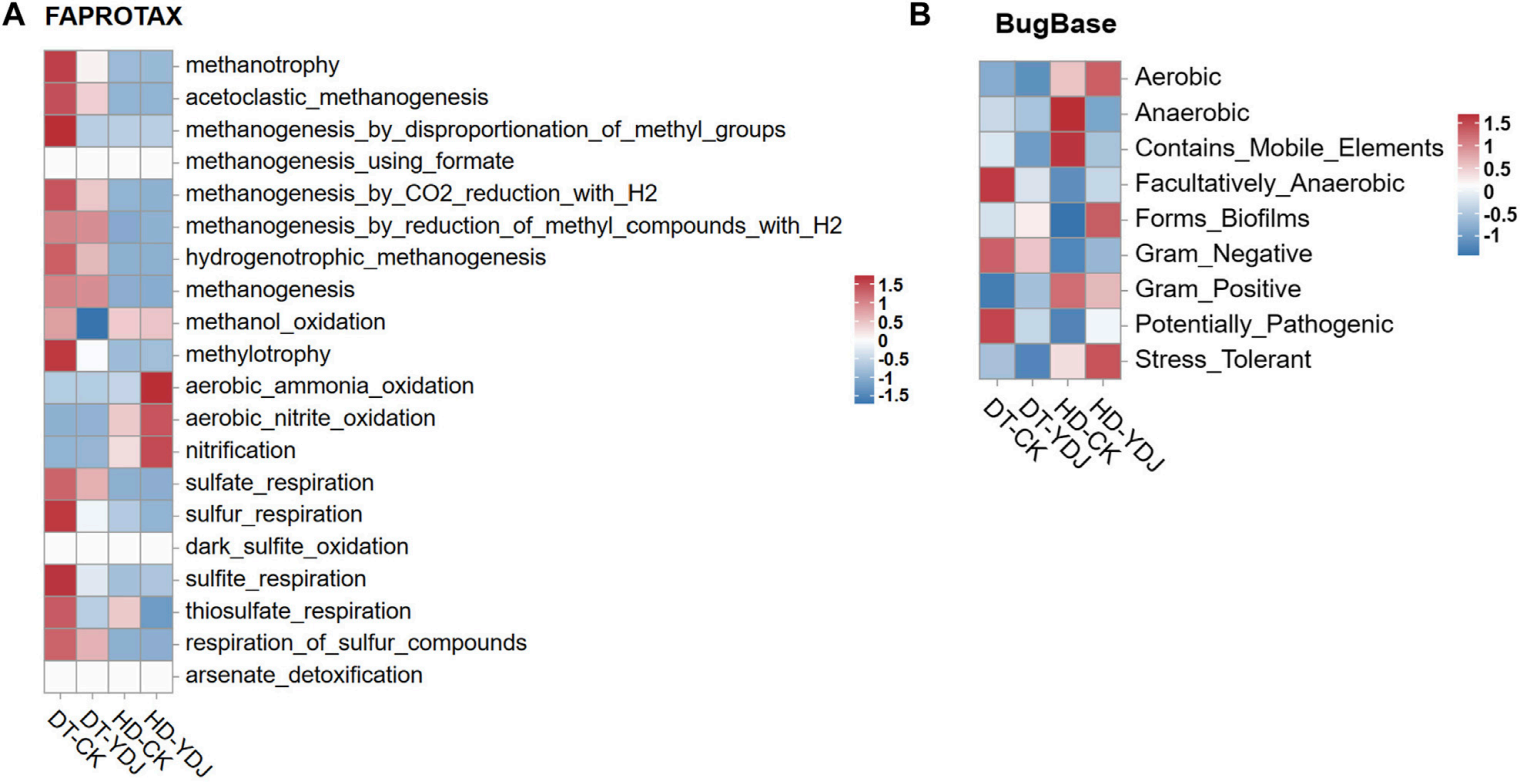
Functional annotation of soil bacteria in DT and HD under ME cultivation. **(A)** shows the FAPROTAX database annotation results, and **(B)** shows the BugBase database annotation results. The horizontal axis indicates the soil groups, the vertical axis indicates the functions, and the colors indicate the relative abundances of the functions. The legends are shown on the right side of the figures.

To understand the effects of ME cultivation on the ecological functions of soil, we analyzed the functions of soil bacteria using the OTU data. We used the FAPROTAX and Bugbase databases to predict the ecological functions of microbial OTUs identified in the DT-YDJ and HD-YDJ treatments. We found that the abundances of multiple functional modules related to methane synthesis decreased in DT-YDJ soil, including methanotrophy, acetoclastic_methanogenesis, and methanogenesis_by_disproportionation_of_methyl_groups. Rice cultivation produces a large amount of methane, which results in excessive greenhouse gas emissions (Yan et al., 2005); this result indicates that ME cultivation has the potential to reduce the methane emissions of DT soils. The abundances of functional modules related to nitrification were higher in HD-YDJ soil than in HD-CK soil, including aerobic_ammonia_oxidation, aerobic_nitrite_oxidation, and nitrification. Nitrification is the key aerobic process of the biogeochemical N cycle (Koch et al., 2015). These findings indicate that the cultivation of ME in HD soil can promote soil micronutrient cycling. The BugBase database annotations (Figure 5B) were used to predict the biological functions of microorganisms (Ward et al., 2017). The cultivation of ME in DT soils decreased the abundance of the Facultatively_Anaerobic and Potentially_Pathogenic classes. This indicates that the cultivation of ME in DT soil might lower the severity of rice diseases by reducing the abundance of the Potentially_Pathogenic class, and additional studies are needed to confirm this possibility. The cultivation of ME in HD soils increased the abundance of Stress_Tolerant, which suggests that ME cultivation might related to enhance the resistance of corn growth to stress. Overall, ME cultivation had positive effects on the health of both DT and HD soils.

### 3.5 Soil bacterial interaction network

**FIGURE 6:**
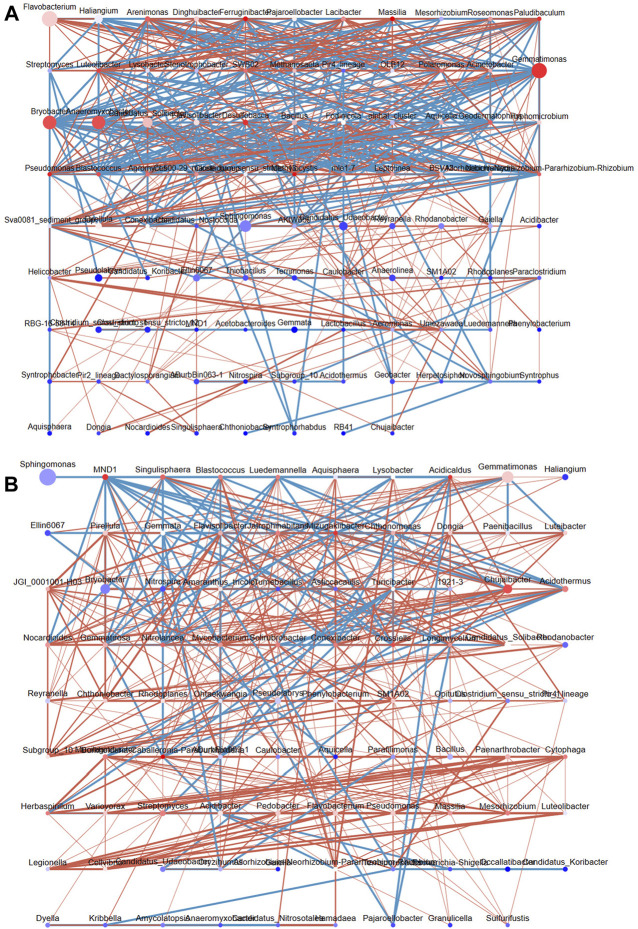
The interaction network of soil bacterial genera in DT and HD under ME cultivation. **(A)** shows the network for DT soils, and **(B)** shows the network for HD soils. The nodes correspond to bacterial genera, with larger nodes indicating higher relative abundance. The color of the nodes ranges from red to blue, with red indicating greater connectivity and blue indicating lower connectivity. The lines indicate correlations, with red indicating positive correlations and blue indicating negative correlations.

We mapped the bacterial interaction network according to the Pearson correlation coefficients to clarify the interactions between bacterial genera in DT and HD soils (Figure 6). In DT soils, the network comprised 96 nodes, and three bacterial genera with high abundance and high connectivity (abundance >2% and connectivity >20) were identified, including *Gemmatimonas*, *Bryobacter*, and *Anaeromyxobacter* (Figure 6A). ME cultivation might affect the abundances of bacterial genera in DT soil, which in turn affects the distribution of soil bacterial diversity. Members of the genus *Gemmatimonas* are capable of anaerobic photosynthesis (Mujakić et al., 2022); however, their effects on soil chemistry remain unclear. The high abundance and connectivity of *Gemmatimonas* in anaerobic rice soil might indirectly affect soil health by affecting other bacteria. The genus *Bryobacter* shows chemoorganotrophic activity and can use sugars, polysaccharides, and organic acids as energy sources; it thus plays a key role in soil metabolism (Pertile et al., 2021). *Anaeromyxobacter* can perform N fixation in soil (Masuda et al., 2020). The network in HD soils consists of 89 nodes (Figure 6B); no bacterial genera with high abundance and high connectivity were detected, and only *Chujaibacter* was found to have a moderate level of connectivity and abundance (abundance >1% and connectivity >10). ME cultivation in DT and HD soils might affect the abundances of these bacteria and thus soil bacterial diversity and health.

### 3.6 Analysis of the correlations between soil chemical properties and bacterial diversity

**FIGURE 7:**
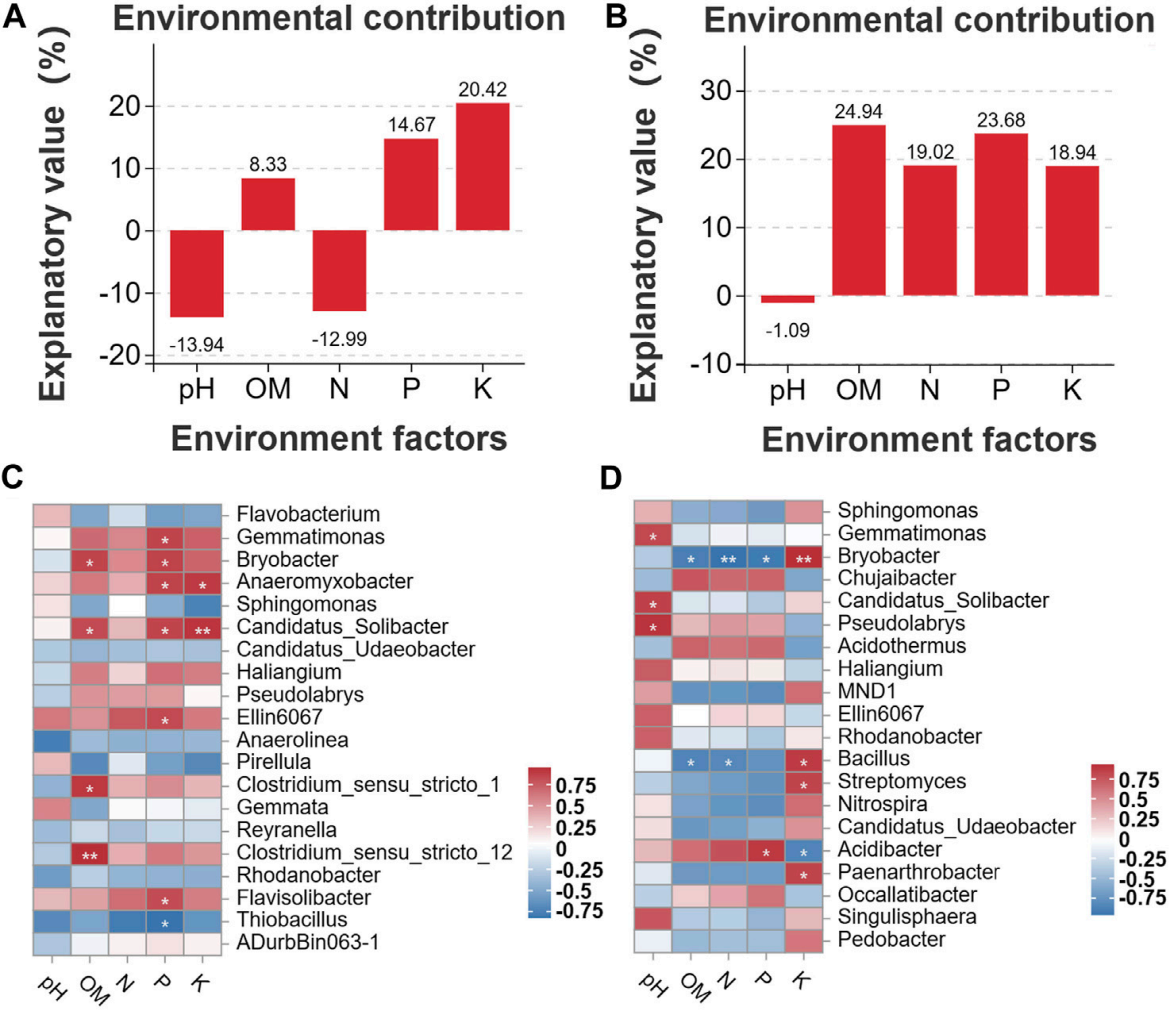
Analysis of correlations between soil chemical properties and bacterial diversity. **(A)** shows the environmental contributions of different factors in DT soil, and **(B)** shows the environmental contributions of different factors in HD soils. The horizontal axis shows the environmental factor, and the vertical axis shows the contribution. Higher percentages indicate greater effects of the environmental factor on the distribution of species. Percentages below 0 indicate no effect. **(C, D)** show the Pearson correlation between soil chemical factors and soil bacterial genera in DT and HD soils. The vertical axis shows soil bacterial genera, and the horizontal axis shows the different soil chemical factors. The numerical value indicates the R-value. “*” corresponds to *p* < 0.05; “**” corresponds to *p* < 0.01; and “***” corresponds to *p* < 0.001.

Correlation analyses were conducted to clarify the associations between the distribution of microbes and the content of soil chemicals. First, we analyzed the environmental contributions of soil chemical factors in DT and HD soils to soil microbial diversity following ME cultivation. The contribution rates of P and K in DT soil were high (contribution rate >10%) (Figure 7A); the contribution rates of OM, N, P, and K in HD soil were also high (contribution rate >10%) (Figure 7B). In DT soil, the abundances of *Gemmatimonas*, *Bryobacter*, *Anaeromyxobacter*, *Candidatus*_*Solibacter*, *Ellin6067*, and *Flavisolibacter* were significantly correlated with the content of P, and the abundances of *Anaeromyxobacter* and *Candidatus*_*Solibacter* were significantly correlated with K (Figure 7C). In HD soil, the abundance of *Acidibacter* was significantly correlated with P, and the abundances of *Bryobacter, Bacillus, Streptomyces*, and *Paenarthrobacter* were significantly correlated with K (Figure 7D). The abundances of the aforementioned bacterial genera are likely affected by the transformation and accumulation of soil chemical properties. Some of these bacterial genera repeatedly appear in the bacterial interaction network (Figure 6), such as *Gemmatimonas*, *Bryobacter*, and *Anaeromyxobacter*, which emphasizes their importance in regulating soil chemistry processes following ME cultivation. We also identified some bacterial genera that were significantly correlated with the accumulation of K following ME cultivation in HD soil. No studies currently indicate that there is a correlation between the abundance of *Bryobacter* and the accumulation of K in soil. Thus, further exploration of the mechanism underlying the effects of this bacterial genus on the accumulation of K in soil is needed to maximize the utilization of this bacterial resource and enhance soil health. *Bacillus* can stimulate the dissolution of soil K and mediate the absorption of K by plants (Sheng, 2005; Ali et al., 2021); ME cultivation in cornfields might increase the content of soil K through the enrichment of *Bacillus. Streptomyces* is the most abundant and arguably the most important actinomycete genus; it is also a rich source of bioactive compounds, antibiotics, and extracellular enzymes (Olanrewaju and Babalola, 2019). *Streptomyces* might regulate the content of soil K through its interactions with soil. Members of the genus *Paenarthrobacter* can play important roles in soil metabolism, as they can degrade various soil pollutants such as herbicides (Zhao et al., 2022). Its metabolic processes might also be related to the accumulation of K, but this possibility needs to be experimentally verified. In general, ME cultivation might affect the abundances of these bacterial genera, and these bacteria might play important roles in regulating soil chemical properties. Additional studies of the interaction between these bacteria and soil are needed. There is a special need for analyses of strains that play a major role in soil K accumulation in *Bryobacter, Bacillus, Streptomyces*, and *Paenarthrobacter*; isolation of these taxa could aid the development of new biofertilizers.

## 4 Conclusion

The effects of ME cultivation on the soil chemical properties and bacterial diversity in DT and HD soils were revealed in this study. The results of our study indicate that this cultivation model has the potential to be widely applied in light of its positive effects on the soil environment and microbial ecology.

## Data Availability

The original contributions presented in the study are publicly available. The raw amplicon sequencing dataset of metabarcoding is available in the NCBI Sequence Read Archive (https://www.ncbi.nlm.nih.gov/sra) under BioSample accession number PRJNA1005455.
