# Supplementary figures and images for: Morchella esculenta cultivation in fallow paddy fields and drylands affects the diversity of soil bacteria and soil chemical properties
Source: Front Genet. 2023 Sep 12;14:1251695. doi: 10.3389/fgene.2023.1251695 (PMC10523323; doi:10.3389/fgene.2023.1251695)

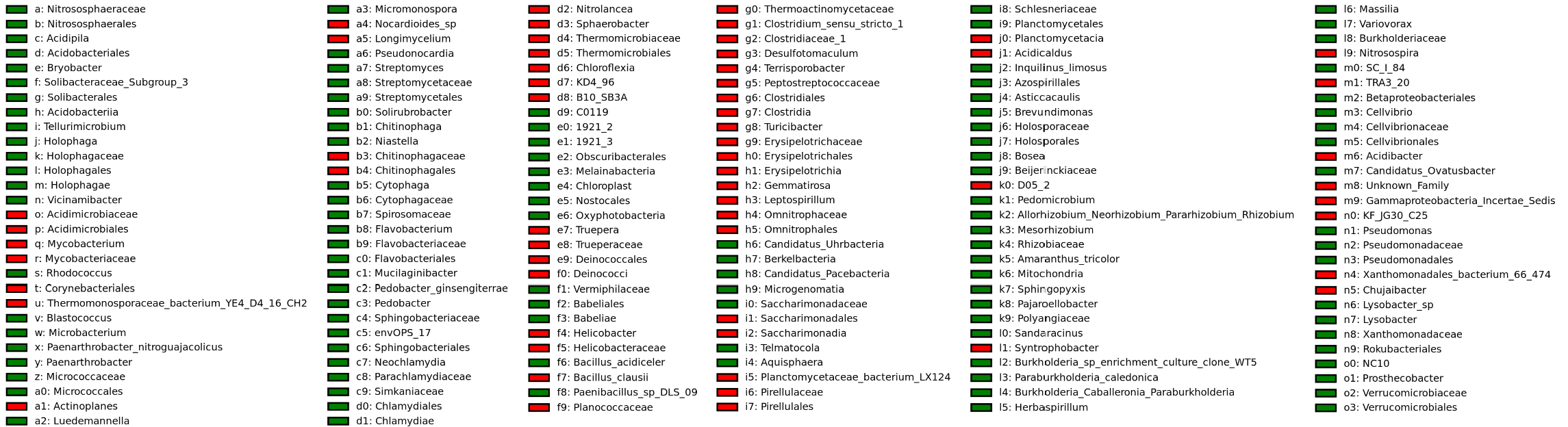

Supplement: Supplementary file 1 [file Image2.PNG]

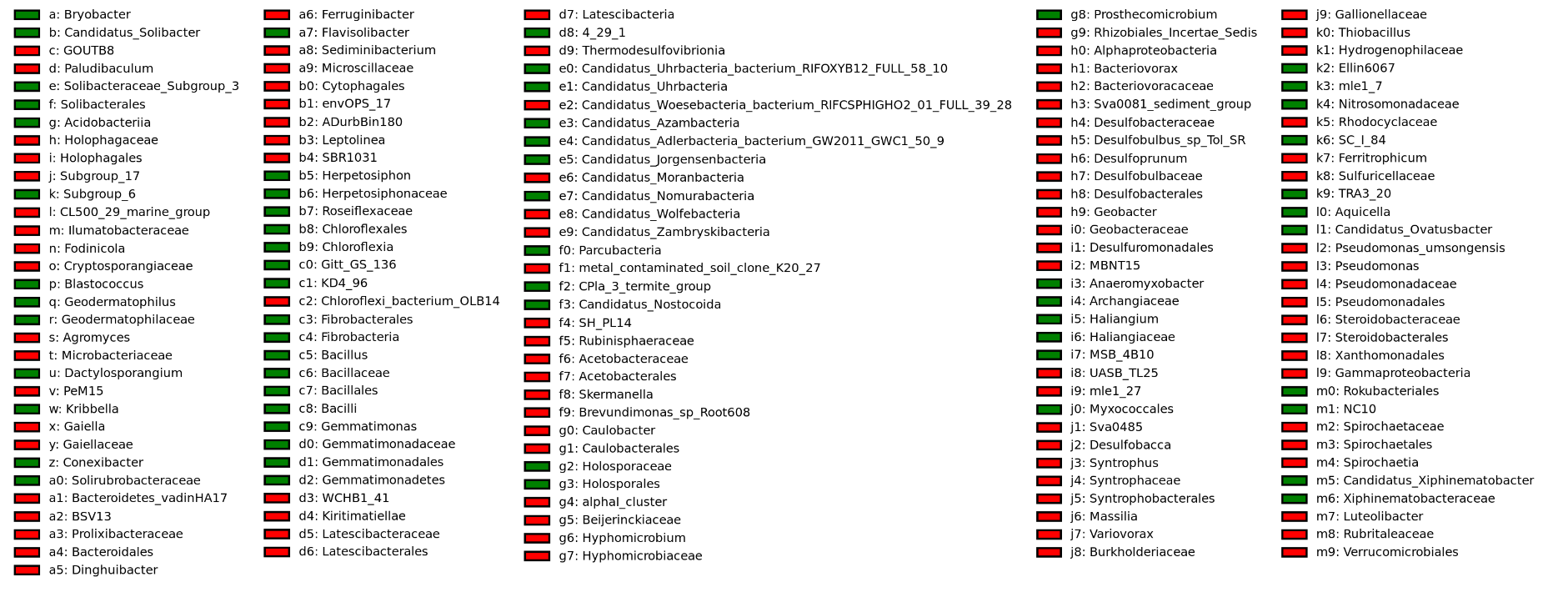

Supplement: Supplementary file 2 [file Image1.PNG]
